# Prevalence of Celiac Disease in Turkish Children with Idiopathic Epilepsy

**Published:** 2014-06

**Authors:** Sedat Işikay, Şamil Hizli, Kutluhan Yilmaz

**Affiliations:** 1 Department of Pediatric Neurology, Gaziantep Children’s Hospital; 2 Department of Pediatric Gastroenterology, School of Medicine, Gaziantep University, Gaziantep, Turkey

**Keywords:** Celiac Disease; Epilepsy; Occipital Paroxysms

## Abstract

***Objective:*** This study has examined the prevalence of celiac disease in Turkish children with idiopathic epilepsy.

***Methods:*** Children with idiopathic epilepsy were screened for celiac disease using the IgA anti-tissue transglutaminase antibody and compared with the healthy control group in order to find the association of celiac disease (CD) with idiopathic epilepsy. Upper gastrointestinal endoscopy and small intestinal biopsies were offered to all antibody-positive patients.

***Findings*** ***:*** A total of 214 children with the diagnosis of idiopathic epilepsy and 166 healthy children as control group were studied. Of the patients recruited, 55.1% had generalized epilepsy, and 44.9% had partial epilepsy. In 33 patients with partial epilepsy, electroclinical features were consistent with a diagnosis of childhood partial epilepsy with occipital paroxysms (CPEO). Two of 33 patients with CPEO had positive IgA anti-tissue transglutaminase antibodies in serology. Pathological examination of small intestinal biopsy specimens showed total villous atrophy in both of them. The prevalence of celiac disease among children with idiopathic epilepsy and CPEO was 0.9% and 6%, respectively.

***Conclusion:*** The results of the present study revealed that prevalence of CD is increased in children with epilepsy. On the other hand, as high as 6% prevalence of CD among patients with CPEO found in this study should be kept in mind and the clinicians should be aware of this association.

## Introduction

Celiac disease (CD) is a chronic autoimmune disease seen in genetically predisposed individuals that is associated with gluten-containing cereals including wheat, rye and barley. Neurological conditions such as cerebellar ataxia, polyneuropathy, headache and epilepsy have been reported in patients with CD^[^^1^^-^^3^^]^. The prevalence of CD among cases with epilepsy is reported within the range of 0.5–9.1%^[^^4^^-^^9^^]^. However, since these studies are generally small-scale, they are far from providing a definitive overview of celiac frequency in children with epilepsy. Immunoglobulin A (IgA) anti-tissue transglutaminase is determined to be highly sensitive (93.1%) and speciﬁc (96.3%) for the diagnosis of CD but the diagnosis^[^^10^^]^ should be conﬁrmed via mucosal biopsy and histological examination.

 Screening CD among epilepsy patients is important in order to prevent long-term complications of CD. On the other hand, the protective role of gluten-free diet on autoimmune disorders and seizure control have been suggested^[^^11^^-^^12^^]^. 

 In this study, we aimed to include a sufficiently large patient population, giving a more reliable estimation of prevalence of celiac disease in epileptic Turkish children.

## Subjects and Methods

***Patients and Control Group***

A total of 214 children with idiopathic epilepsy were studied over a period of one year (2011–2012). None of the subjects had any complaints related to gastrointestinal tract or a suspicion of CD. Neurological examination and intellectual level were normal in all patients. The 1989 classification of epilepsy by the International League against Epilepsy was used for the diagnostic classification of study patients^[^^13^^]^. Children with secondary epilepsy (involving cerebral malformations, metabolic disorders, infections, head injury, tumors, or cerebral palsy) were excluded from the study. The control group comprised 166 children age and sex matched with the study group. They had no gastrointestinal and neurologic disorders and admitted to the Gaziantep University Hospital, Department of Pediatrics for various reasons, such as upper respiratory tract infections. Medical history of all control children was recorded and neurologic status was evaluated.

 The study was approved by the Ethics Committee of Gaziantep University Faculty of Medicine. Informed consent was obtained from the parents of all children. All the subjects were screened for IgA anti-tissue transglutaminase (tTG) antibody and serum IgA analysis. Subjects with confirmed positive tTG antibody were offered an endoscopic small intestinal biopsy. Biopsy specimens were assessed according to a modified Marsh classification^[^^14^^]^. 

***Laboratory Methods***

A commercially available microplate enzyme-linked immune sorbent assay (Euroimmune, GmbH, Lübeck, Germany) was used to test for IgA tTG. The threshold for a positive assay result was set as 20 RU/ml. According to the ESPGHAN guideline for diagnosis of CD, a value of 10× the upper-normal level was accepted as diagnostic^[^^15^^]^. To confirm the diagnosis of CD, four mucosal biopsies were taken endoscopically from multiple sites of the second part of the duodenum (Olympus GIF P230 videogastroscope, Olympus Optical Corporation, Tokyo, Japan).

***Statistical Analysis***

In Turkey, the prevalence of biopsy-proven celiac disease in the normal healthy children was reported as 0.47%^[^^16^^]^. Since previous studies in Turkey had found that the highest CD frequency within an epilepsy group was 9.1%, we estimated a 5% frequency in our study population and determined the minimum study group size as 191 patients with 80% minimum power. A total of 214 patients who were admitted to our clinic during the one-year period of this study were included in the study.

 Statistical analyses were performed with SPSS for Windows (version 11.0; SPSS Inc., Chicago, IL). χ^2^ and Student t-test were used for group comparisons. A *P*-value of <0.05 was considered as statistically significant.

***Findings***

A total of 214 children with epilepsy (112 boys, 102 girls; age range: 5 months to 17 years; mean age: 9.33±4.03 years) were studied. All epileptic patients were under anti-epileptic drug treatment. The control group consisted of 166 healthy children (83 boys, 83 girls; age range: 6 months to 17 years; mean age: 9.35±4.75 years). 

 The mean age of the patients and control group was; 9.33±4.03 and 9.35±4.75 years, respectively. In patients group; 112 (52.3%) of the cases were male while 102 (47.7%) of them were female; similarly in control group 83 (50%) of cases were female while other 83 (50%) were male. Patients and control group had no statistically significant difference in terms of age and sex.

 Of the 214 patients with epilepsy recruited, 55.1% had generalized epilepsy, and 44.9% had partial epilepsy. In 33 patients with partial epilepsy, the electroclinical features were consistent with a diagnosis of childhood partial epilepsy with occipital paroxysms (CPEO). 

**Fig 1:**
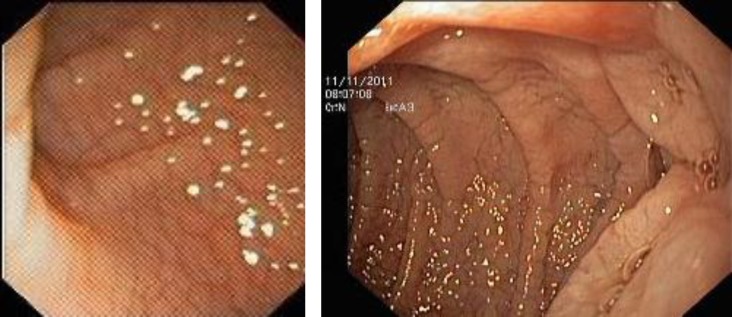
Endoscopic view of distal duodenum of patients with celiac disease showing scalloping of folds and "cracked-mud" appearance to mucosa (a,b).

Two of the 33 patients with CPEO (6%, one girl and one boy, age at onset, 10 and 5 years, respectively) had positive IgA tTG (350 and 348 IU, respectively).

**Fig 2:**
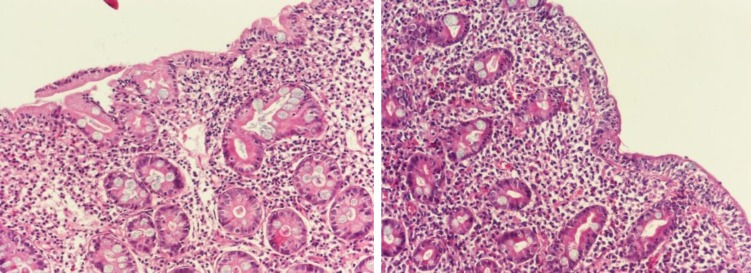
The microscopic views of the distal duodenal biopsy specimens in patients with celiac disease (a,b). Note the characteristic features of blunting of villi, crypt hyperplasia, and increased intraepithelial lymphocytes of crypts (Hematoxylin and eosin stain, 200×).

 Upper gastrointestinal endoscopy (Fig 1) and pathological examinations of duodenal biopsy (Fig 2) showed total villous atrophy (Marsh type 3). These patients were both positive for HLA DQ2. The frequency of biopsy-proven CD was 6% (2/33) in children with CPEO. 

 The prevalence of celiac disease in the children with idiopathic epilepsy was 0.9% (2/214). Hematologic and biochemical investigations including plasma levels of vitamin B_12_, vitamin E, and folate were normal in those patients. Additionally, brain computed tomography and brain magnetic resonance imaging of children with celiac disease revealed no intracranial calcifications. They were on a gluten-rich diet at the time of diagnosis; following diagnosis, both patients have maintained a gluten-free dietary regimen. 

 None of the control cases had positive IgA tTG. All patients and controls had normal serum IgA levels. There was no statistically significant difference in IgA tTG positivity percentages between generalized and focal epilepsy groups (*P*=0.2). There was significant difference in IgA tTG positivity percentage between the CPEO group and others (*P*<0.019). Comparison of generalized epilepsy patients and CPEO groups in terms of IgA tTG antibody positivity also showed statistical significance (*P*=0.007).

## Discussion

In this study we have determined that the prevalence of CD is increased in children with epilepsy; especially in patients with CPEO. Previous studies have reported different rates of CD in epileptic children. Zelnik et al^[^^4^^]^, Kieslich et al^[^^5^^]^, Fois et al^[^^6^^]^., and Ruggieri et al^[^^7^^]^ reported the rates of 7.2%, 1.3%, 1% and 0.5%, respectively. In Turkey, Ertekin et al^[^^8^^]^ and Dalgıç et al^[^^9^^]^ reported 9.1% and 1.17%, respectively. The rate of CD in the present study was 0.93%, indicating a two-fold increase of CD among patients diagnosed with epilepsy compared with the 0,47% of prevalence of CD among healthy children in Turkey^[^^16^^]^. Similarly, Pratesi et al^[^^17^^]^, found this rate as 2.3. Additionally, Labate et al^[^^19^^]^, reported CD in 8% of CPEO patients. Similarly, we found CD in 6% of patients with CPEO.

 In contrast, the prevalence of biopsy-proven CD in children with idiopathic epilepsy was not significantly higher compared to controls (0.8% vs.0.6%) in a study from Serbia^[^^18^^]^. However, there was not a CPEO group among cases which may be affecting the results in this study. 

 The cause of association between celiac disease and epilepsy is not clearly understood. Suggested autoimmune mechanisms concentrate on vasculitis and vitamin deficiency. Identification of antitumoral and antiganglioside antibodies in celiac patients with neurologic disorders, and clinical improvement with antibody loss in some cases by early start of gluten-free diet suggest that neurologic disorders can be caused by antibody-mediated autoimmune mechanisms^[^^1^^,^^2^^,^^20^^,^^21^^]^. Accordingly, antibodies against gliadin may cause production of similar antibodies against brain tissue, leading to neurotoxicity. However, previous studies did not show antibodies against brain tissue. Pratesi et al^[^^17^^]^ only indicated the formation of antibodies against brain blood vessels via immune fluorescence method. Hadjivassiliou et al^[^^22^^]^ reported that neurologic symptoms could appear due to neurotoxic effect of gliadin without celiac disease development.

 Occipital lobe epilepsy is a relatively rare form of focal epilepsy and paroxysmal visual manifestations are the hallmark of epileptic seizures arising from the occipital lobe^[^^23^^]^. Gobbi et al^[^^24^^]^, defined association of epilepsy, cerebral calcification and celiac disease as CEC syndrome, and found that 61% of their cases with CD and cerebral calcifications had occipital epilepsy. Central nervous system folate deficiency has been suggested to have a possible role in this syndrome. Some of these patients have been determined to be difficult to treat and to develop drug resistance^[^^24^^]^. Seizure control may be improved with the institution of a gluten free diet with folic acid supplements in those patients^[^^19^^,^^25^^]^.

 Occipital calcifications^[^^24^^]^ and focal white matter changes^[^^5^^]^ were observed in central nervous system imaging of patients with CD. In our study, central imaging of two patients showed no pathology. The observation that central involvement was not reported in similar studies in Turkey but was prevalent in European countries like Italy suggests that racial and individual differences may be associated with central calcification.

## Conclusion

The results of the present study revealed that prevalence of CD is 0.93% in children with epilepsy. However screening CD in all patients with epilepsy is still not cost-effective and practical. On the other hand, as high as 6% prevalence of CD among patients with CPEO found in this study, should be kept in mind and the clinicians should be aware of this association. With this finding we can conclude that patients diagnosed with CPEO should be screened for CD. Further studies are warranted to show the difference in the prevalence of CD between patients with CPEO and those with other types of epilepsy. More importantly, if CD is more common among patients with CPEO, the effects of gluten-free diet on CPEO should also be investigated.

## Authors’ Contribution

Concept / Design: S. Işıkay 

Acquisition of Data: S. Işıkay

Data Analysis / Interpretation; S. Işıkay, Ş. Hızlı

Manuscript Preparation: S. Işıkay, Ş. Hızlı, K. Yılmaz

Critical Revision of the Manuscript: S. Işıkay, Ş. Hızlı, K. Yılmaz

All authors approved the final version of the aticle.
